# MeningiSSS: A New Predictive Score to Support Decision on Invasive Procedures to Monitor or Manage the Intracerebral Pressure in Children with Bacterial Meningitis

**DOI:** 10.1007/s12028-019-00792-7

**Published:** 2019-07-24

**Authors:** Urban Johansson Kostenniemi, Linda Karlsson, Sven-Arne Silfverdal, Christer Mehle

**Affiliations:** 1 Department of Clinical Sciences, Pediatrics, Umeå University, 901 87 Umeå, Sweden; 2 Department of Clinical Microbiology, Infectious Diseases, Umeå University, Umeå, Sweden

**Keywords:** Bacterial meningitis, Decision support techniques, Disease management, Risk factors, Risk assessment

## Abstract

**Background:**

Knowing the individual child’s risk is highly useful when deciding on treatment strategies, especially when deciding on invasive procedures. In this study, we aimed to develop a new predictive score for children with bacterial meningitis and compare this with existing predictive scores and individual risk factors.

**Methods:**

We developed the Meningitis Swedish Survival Score (MeningiSSS) based on a previous systematic review of risk factors. From this, we selected risk factors identified in moderate-to-high-quality studies that could be assessed at admission to the hospital. Using data acquired from medical records of 101 children with bacterial meningitis, we tested the overall capabilities of the MeningiSSS compared with four existing predictive scores using a receiver operating characteristic curve (ROC) analysis to assert the area under the curve (AUC). Finally, we tested all predictive scores at their cut-off levels using a Chi-square test. As outcome, we used a small number of predefined outcomes; in-hospital mortality, 30-day mortality, occurrence of neurological disabilities at discharge defined as Pediatric Cerebral Performance Category Scale category two to five, any type of complications occurring during the hospital stay, use of intensive care, and use of invasive procedures to monitor or manage the intracerebral pressure.

**Results:**

For identifying children later undergoing invasive procedures to monitor or manage the intracerebral pressure, the MeningiSSS excelled in the ROC-analysis (AUC = 0.90) and also was the only predictive score able to identify all cases at its cut-off level (25 vs 0%, *p* < 0.01). For intensive care, the MeningiSSS (AUC = 0.79) and the Simple Luanda Scale (AUC = 0.75) had the best results in the ROC-analysis, whereas others performed less well (AUC ≤ 0.65). Finally, while none of the scores’ results were significantly associated with complications, an elevated score on the MeningiSSS (AUC = 0.70), Niklasson Scale (AUC = 0.72), and the Herson–Todd Scale (AUC = 0.79) was all associated with death.

**Conclusions:**

The MeningiSSS outperformed existing predictive scores at identifying children later having to undergo invasive procedures to monitor or manage the intracerebral pressure in children with bacterial meningitis. Our results need further external validation before use in clinical practice. Thus, the MeningiSSS could potentially be helpful when making difficult decisions concerning intracerebral pressure management.

## Introduction

Since children with bacterial meningitis often have symptoms far from typical, predictive tools designed to identify severe infections have been developed and successfully implemented in clinical practice [1]. Despite knowing several individual risk factors associated with adverse outcomes [2], no tool aimed at predicting complications or outcome has achieved widespread clinical use. This is mainly due to difficult criteria or inconsistent results in external validations [3–10]. As a result, there is a lack of accurate tools for facilitating treatment decisions such as the use of invasive procedures.

Mortality rates for bacterial meningitis in children are high; around 4–10% in high-income countries and even higher in low- and middle-income countries [11–13]. In fatal cases, death can occur due to complications of circulatory failure or due to intracerebral injury. In addition to death, intracerebral injury can also cause long-term neurological disabilities such as impaired hearing, epilepsy, and cognitive defects, affecting up to one-third of survivors [11–15].

To provide the best possible care, treatment guidelines offer recommendations regarding antibiotics, corticosteroids, and the use of intensive care [11–13, 16, 17]. The major challenge, however, is identifying and managing rapidly increasing intracerebral pressure. This is important because the inflammatory response to the infection risks cause intracerebral injury and raise the intracranial pressure, resulting in further damage or risk of herniation resulting in death [11–13]. Invasive procedures may be required to monitor or to reduce the intracerebral pressure, including placement of an external ventricular drainage or, in extreme cases, performing a hemicraniectomy [18]. These invasive procedures involve risks and when needed, they must be performed within a few hours, otherwise the intracerebral injury can progress rapidly [18]. In addition, as these are neurosurgical procedures, transfer to another hospital is often required. Estimating the individual child’s risk would be helpful and enable clinicians to make a referral decision early on, rather than when the child’s condition is rapidly deteriorating.

In this study, we aim to develop a new predictive score for children with bacterial meningitis and compare this with existing predictive scores and individual risk factors.

## Methods

In this retrospective study, we developed a new predictive score for children with bacterial meningitis, the “Meningitis Swedish Survival Score” (MeningiSSS), based on a systematic review [2]. We then used a database containing 101 cases of bacterial meningitis [1, 19] to test our score’s predictive ability for a limited number of predefined outcomes and to compare it with existing predictive scores and individual risk factors (Table 1).

**Table 1:** Criteria of the MeningiSSS and existing predictive scores

Predictive scores	
*Aronin Scale* [3]: *high risk if two points or more*	
Systolic blood pressure ≤ 90 mmHg or > 40 mmHg decrease (1p)^a^	Altered mental status (1p)
	Seizures (1p)
*Herson–Todd Scale* [4, 5]:* high risk if 4.5 points or more*	
Coma (3p)	CSF WBC count < 1000 cells/µL (1p)
Body temperature < 36.6 °C (2p)	Hemoglobin < 110 g/L (1p)
Seizure (2p)	CSF glucose < 1.1 mmol/L (0.5p)
Systolic blood pressure < 60 mmHg (1p)^a^	Symptoms more than 3 days (0.5p)
Age under 12 months (1p)	
*Meningitis Swedish Survival Score (MeningiSSS): high risk if six points or more*	
Altered consciousness (2p)	Respiratory distress^d^ (1p)
Seizures (2p)	Fever more than seven days (1p)
Low blood WBC count^b^ (2p)	CSF WBC count < 1000 cells/µL (1p)
Circulatory distress, either circulatory shock (2p)^b^ or peripheral circulatory failure (1p)^c^	CSF glucose level ≤ 0.6 mmol/L (1p)
	CSF protein level ≥ 2500 mg/L (1p)
*Niklasson Scale* [3]:* high risk if three points or more*	
CSF WBC count < 100 cells/µL (1p)	Body temperature > 40 °C (1p)
Systolic blood pressure < 100 mmHg (1p)^a^	Blood WBC count < 15 × 10^9^/L (1p)
Petechiae less than 12 h (1p)	Platelet count < 100 x 10^9^/L (1p)
*Simple Luanda Scale* [5]:* high risk if seven or more (adverse outcomes), or, eight points or more (death)*	
No electricity at home (2p)	Seizures (1p focal, 2p if general)
Symptoms for four to 7 days (1p), or more than 8 days (2p)	Altered consciousness (5p) or coma (10p)
	Dyspnoea (1p if moderate, 2p if severe)^e^

This table shows the scoring criteria for the predictive scores tested in our study

*CSF* cerebrospinal fluid, *WBC* white blood cells

^a^If blood pressure at admission was missing, the criteria were deemed fulfilled if the patient needed inotropic drugs to maintain circulation

^b^According to the age-correlated systemic inflammatory response syndrome (SIRS) criteria [31]

^c^Hypotension according to the age-correlated systemic inflammatory response syndrome (SIRS) criteria [31] or clinical signs of peripheral circulation failure

^d^Tachypnoea according to the age-correlated systemic inflammatory response syndrome (SIRS) criteria [31] or need of respirator

^e^Moderate dyspnoea were defined as tachypnoea according to the age-correlated systemic inflammatory response syndrome (SIRS) criteria [31] and severe dyspnoea were defined as need of respirator

### Creating the Meningitis Swedish Survival Score (MeningiSSS)

We based our predictive score on a systematic review of 31 individual studies on risk factors for adverse outcomes in children with bacterial meningitis [2]. The systematic review, using a revised QUIPS list for scoring methodological quality of prognosis studies, graded the studies as either low, moderate, or high quality. From this, we selected risk factors identified in moderate-to-high-quality studies that were related to any type of adverse outcome and could also be assessed at admission to the hospital either already at the emergency department or during the first day of care. Each risk factor was then given a numeric value in relation to its relative risk increase. Factors increasing risk of adverse outcomes by 100% were given a value of one, and factors increasing risk by 200% a value of two. Finally, we decided that the score was to be calculated by adding the values of all risk factors that the child fulfilled (Table 1).

### Database

During two previous studies [1, 19], we created a database containing information on 1143 cases of severe infections in children aged one month to 17 years being treated in Västerbotten County, Sweden, during the period 1986–2015. Cases for this database were identified using Västerbotten County Council’s diagnosis registry searching for international classification of disease diagnostic (ICD) codes of bacterial meningitis and sepsis (ICD-8/9: 036, 038 and 320, ICD-10: A39, A40, A41 and G00), as well as laboratory records of positive bacterial cultures.

To ensure all cases in the database were correct, all identified cases underwent a validation process before being included in the database. For bacterial meningitis, at least two of the following criteria had to be fulfilled:

1. ICD diagnosis of bacterial meningitis (ICD-8/9: 036 and 320, ICD-10: A39 and G00);
2. Positive cerebrospinal fluid culture or;
3. Clinical presentation consistent with bacterial meningitis using the Bacterial Meningitis Score [20] or the clinical decision rule as stated by Oostenbrink et al. [21].

### Inclusion and Exclusion Criteria for this Study

From the database, we included cases of children aged one month to 17 years validated as bacterial meningitis. We excluded any postoperative neurosurgical infections. In addition, cases with missing variables were excluded from analyses of a specific predictive score if more than one criterion were missing in a score based on five criteria or less, or more than two criteria in a score based on more than five criteria.

### Variables

The database already contained information on previous illnesses and clinical signs, symptoms and laboratory findings registered in the emergency department at admission to the hospital. Using a standardized protocol, we reviewed medical records and added information on all pharmaceutical treatments and invasive procedures, results of any tests, and any complications occurring during the hospital stay. In addition, we used the Swedish Population Register to assess 30-day mortality.

### Outcomes

Clinical outcome at discharge was retrospectively graded for all included children using the Pediatric Cerebral Performance Category Scale (PCPC) based on a clinical assessment by the discharging physician. The PCPC scale grades neurological disability and functions into six categories, where category one represents a fully normal functioning level, category two to four mild-to-severe neurological disabilities, category five children in coma or vegetative states, and category six represents diseased children [22].

We used three primary outcomes: in-hospital mortality and 30-day mortality, both equivalent to PCPC category six, and occurrence of neurological disabilities at discharge defined as PCPC category two to five. These were tested separately and in combination defined as “any adverse short-term outcome,” equivalent to PCPC category two to six.

We also used three secondary outcomes: any type of complications occurring during the hospital stay, use of intensive care, and use of invasive procedures to monitor or manage the intracerebral pressure. The latter included placement of devises for either continuously measuring the intracerebral pressure or draining cerebrospinal fluid to reduce the intracerebral pressure. In addition, any neurosurgical procedures aimed at reducing the intracerebral pressure were also registered.

### Selection of Comparison Scores

To identify possible predictive scores for comparison, we conducted a systematic search on PubMed for studies published until December 31, 2018, that included the term “meningitis” together with either “predict*” or “outcome” in the title. Of the 498 publications matching our search criteria, 56 publications had a relevant title and their abstracts were read. From these, we identified 18 publications focusing on scoring systems for predicting outcome in cases of bacterial meningitis. When reading these publications in full, another 19 publications were identified from reference lists, and these were also read in full. In total, 18 different scoring systems were identified using this method. Of these, four were later selected for comparison based on results in previous studies and assessability at admission to the hospital; the Aronin Scale, the Herson–Todd Scale, the Niklasson Scale and the Simple Luanda Scale (Table 1). In addition, the Glasgow Coma Scale being part of the MeningiSSS was also included, whereas two other scoring systems based on neurological function were not since they required specific neurological examinations that had not been performed on the children in our retrospective material [6]. Three scoring systems were excluded due to being specific to patients already receiving intensive care since the aim of this study was to create a predictive score based on criteria assessable at admission to the hospital [7]. The remaining six predictive scores had either underperformed in previous studies, or not excelled despite being based on complicated mathematical algorithms [3–10].

### Statistics

We performed all statistical analyses in IBM SPSS Version 24 (IBM Corp.). First, we used the Chi-square test for percentages and Levene’s test of equality combined with a *t* test for means, presented with either a *p* value or a 95% confidence interval. In the same manner, the Chi-square test was used to compare all predictive scores at their respective cut-off level. Finally, we performed a receiver operating characteristics (ROC) curve analysis to calculate the area under the curve (AUC) for each predictive score to test overall ability and fit. Based on AUC levels, the results were graded into the following previously validated performance categories; “excellent” (AUC ≥ 0.9), “good” (AUC ≥ 0.8), “fair” (AUC ≥ 0.7), “poor” (AUC ≥ 0.6), and “failed” (AUC < 0.6) [23].

### Ethics

Our study was approved by the Regional Ethics Board in Umeå (ethical approval numbers; 08-208 M, 2015/336-32 and 2017/182-31).

## Results

Of the 1143 cases of severe infections in the database, 101 matched all criteria and were thereby included in our study. These cases were distributed on 97 children, of whom three children with predisposing conditions had multiple episodes of bacterial meningitis (Table 2).

**Table 2:** General features and clinical presentation at admission

	All patients	Nos.
Patient characteristics		
Age at admission (years and months)	4y 3 m (3:2–5:5)	[101]
Girls (%)	47 (37–56)	[101]
Boys (%)	54 (44–63)	[101]
Known predisposing conditions (%)	29 (20–38)	[101]
Symptoms and clinical findings		
Seizures (%)	11 (6–18)	[101]
Neck stiffness (%)	51 (41–60)	[101]
Altered mental status (%)	65 (56–74)	[101]
Septic appearance (%)^a^	70 (60–78)	[99]
Laboratory findings		
C-reactive protein (mg/L)	153 (127–179)	[93]
WBC in blood (10^9^/L)	14.9 (13.1–16.6)	[90]
Cerebrospinal fluid results		
Protein (mg/L)	2091 (1798–2384)	[66]
Lactate (mmol/L)	20,8 (0.0–45.7)	[70]
Glucose (mmol/L)	2,0 (1.5–2.5)	[65]
WBC (cells/µL)	4044 (2657–5433)	[86]
Poly (cells/µL)	3287 (2041–4532)	[85]
Mono (cells/µL)	795 (439–1150)	[84]
Causative pathogen (%)		
*H. influenzae*	41 (31–50)	[101]
*S. pneumoniae*	32 (22–41)	[101]
*N. meningitidis*	11 (5–17)	[101]
Another identified pathogen^b^	8 (3–13)	[101]

This table shows the general features and clinical presentation at admission for the patients included in our study. Occurrence presented as percentages and averages as means followed by 95% confidence interval presented inside parenthesis. The number of included patients for each analysis is stated in brackets

*WBC* white blood cells

^a^Fulfilling at least two age-correlated systemic inflammatory response syndrome (SIRS) criteria [31]

^b^This group included four cases of group B streptococci and one case each of group A streptococci, *salmonella*, *Echinacea coli*, and *Mycoplasma pneumoniae*

All 101 included cases had been given an ICD diagnosis of bacterial meningitis. Of these, 73 were verified by a positive cerebrospinal culture. In the remaining 28 cases verified by using the Bacterial Meningitis Score [20], the cerebrospinal fluid was positive in a polymerase chain reaction test or antigen test in 11 cases and positive in direct microscopy for bacteria in an additional five cases. Of the remaining 12 cases, eight had cerebrospinal fluid analyses consistent with bacterial meningitis in combination with typical symptoms and clinical findings. Finally, in the last four cases, the children all had fever and a C-reactive protein elevated at 252–410 mg/L in combination with neurological symptoms and either neck stiffness or a bulging fontanel.

During the hospital stay, almost half of the children were treated at an intensive care unit. Furthermore, ten children underwent an invasive procedure to monitor or manage the intracerebral pressure (Table 3); an intracerebral pressure monitor was placed in seven of these cases, this was later converted to an external ventricular drain due to rising intracerebral pressure in four cases. In addition, an external ventricular drain was placed directly without previous use of an intracerebral pressure monitor in an additional three cases. No child underwent a hemicraniectomy or any other type of neurosurgical procedure aimed at reducing the intracerebral pressure.

**Table 3:** Treatment strategies during the hospital stay

Treatment strategies	All patients	Nos.
Initial antibiotic treatment (%)		
Cephalosporins	92 (85–96)	[99]
Carbapenems	3 (1–8)	[99]
Addition of ampicillin	31 (23–41)	[99]
Duration of antibiotic treatment (days)	12 (11–13)	[99]
Addition of corticosteroids (%)^a^	68 (58–76)	[99]
Intensive care (%)	49 (39–59)	[100]
Invasive intracranial pressure monitoring or management^b^ (%)	10 (5–17)	[101]
Duration of hospital stay (days)	14 (13–16)	[91]

This table shows treatment strategies during the hospital stay for the patients included in our study. Occurrence presented as percentages and averages as means followed by 95% confidence interval presented inside parenthesis. The number of included patients for each analysis is stated in brackets

^a^Corticosteroids were administered just before or simultaneously with the first antibiotic dosage in all cases where corticosteroids were added

^b^This category included use of either an intracerebral pressure monitor or an external ventricular drain

Complications occurred during the hospital stay in 50% (CI 40–60) of all cases. Besides intracerebral structural complications presented specifically, these complications included transient neurological disabilities or repeated seizures, Syndrome of Inappropriate Anti-Diuretic Hormone secretion, renal failure, osteomyelitis, or septic arthritis, severely affected coagulation including disseminated intravascular coagulation or abdominal organ hemorrhages, hydrocele, partial amputations, and heart arrythmias.

Intracerebral structural injury occurred in 14% (CI 8–22) of all cases. The most common being hygroma occurring in six cases followed by generalized brain edema in three cases, cerebral hemorrhages in three cases, ischemic injury in two cases, and a brain abscess in one case. All intracerebral complications were diagnosed via a brain computer tomography except for three cases: a hygroma diagnosed via repeated brain ultrasounds, an ischemic injury diagnosed using magnetic resonance, and a subarachnoid hemorrhage discovered during postmortem autopsy.

At discharge, a full recovery (PCPC category one) was seen for 78% (CI 69–85) of all children, whereas an adverse short-term outcome (PCPC category two to six) occured in 22% (CI 15–31) either neurological disabilities (PCPC category two to five) seen in 16% (CI 10–24) or death (PCPC category six) seen in 6% (CI 3–12). No additional deaths occurred within 30 days of discharge.

Our new predictive score, the MeningiSSS, was equal or better than existing predictive scores in most analyses. In particular, it excelled at identifying children later having to be admitted to the intensive care unit or undergo invasive procedures to monitor or manage the intracerebral pressure. Multiple cut-off levels were tested to determine which cut-off level had the best discrimination. This was obtained at a cut-off level of six points or higher, which is why this cut-off level was used when comparing the MeningiSSS with the other predictive scores and individual risk factors.

### MeningiSSS Compared with Other Predictive Scores

A high MeningiSSS score on admission was strongly associated with the use of invasive procedures to monitor or manage the intracerebral pressure. When tested using ROC-analysis, the MeningiSSS had an AUC of 0.90 and was thereby graded into the category “excellent” according to the previously validated grading system [23]. In the same analysis, the Simple Luanda Scale and the Niklasson Scale, with an AUC of 0.68 and 0.60, respectively, were both graded as “poor” [23]. Finally, the remaining two predictive scores, both having an AUC below 0.60, were graded as “failed” [23]. At their respective cut-off levels, only the MeningiSSS and the Simple Luanda Scale had statistically significant results (Table 4). Further, in identifying children later having to be admitted to the intensive care unit, the MeningiSSS and the Simple Luanda Scale had the best overall results; ROC-analysis graded both as “fair” with an AUC of 0.79 for the MeningiSSS and an AUC of 0.75 for the Simple Luanda Scale [23]. In the same analysis, the remaining three predictive scores all had an AUC below 0.70 and were thereby graded as “poor” [23]. Again, when testing their cut-off levels, significant differences for use of intensive care were only seen for the MeningiSSS and the Simple Luanda Scale (Table 4).

**Table 4:** Comparison of the predictive scores’ ability to identify children later having to be admitted to the intensive care unit or undergo invasive procedures to monitor or manage the intracerebral pressure

	No.	AUC	Grade	Sens. (%)	Spec. (%)	PPV (%)	NPV (%)	*p*^Cut-off^
Intensive care								
The Aronin Scale	100	0.65	Poor	27	90	28	56	0.03
The Herson–Todd Scale	88	0.64	Poor	14	96	75	54	0.12
The MeningiSSS	77	0.79	Fair	56	88	79	72	< 0.01
The Niklasson Scale	88	0.61	Poor	9	98	80	52	0.17
The Simple Luanda Scale^a^	99	0.75	Fair	42	94	87	63	< 0.01
Invasive ICP monitoring or management^b^								
The Aronin Scale	101	0.59	Failed	30	84	17	92	0.29
The Herson–Todd Scale	88	0.58	Failed	17	92	13	94	0.50
The MeningiSSS	77	0.90	Excellent	100	75	25	100	< 0.01
The Niklasson Scale	88	0.60	Poor	0	94	0	92	0.50
The Simple Luanda Scale^a^	99	0.68	Poor	56	80	22	95	0.02

This table shows the ability of the Meningitis Swedish Survival Score (MeningiSSS) of identifying children later having to be admitted to the intensive care unit or undergo invasive procedures to monitor or manage the intracerebral pressure, compared with existing predictive scores. First, the number of patients included for each predictive score (No.) is shown, followed by the area under the curve (AUC) obtained from the receiver operating curve characteristics analysis together with the performance category grading (Grade) based on this result [23]. Lastly, each predictive score’s sensitivity (Sens.), specificity (Spec.), positive predictive value (PPV), and negative predictive value (NPV) when tested at their cut-off levels using a Chi-square test are shown, including the *p* value of this analysis (*p*^Cut-Off^)

*ICP* intracranial pressure

^a^In this analysis, the Simple Luanda Scale was tested using the cut-off level stated for predicting adverse outcomes

^b^This category included use of either an intracerebral pressure monitor or an external ventricular drain

Concerning association with death and any adverse outcome, the MeningiSSS and the Niklasson Scale had the best overall results. For death (PCPC category six), the MeningiSSS, the Herson–Todd Scale, and the Niklasson Scale with an AUC of 0.70, 0.79, and 0.72 respectively, were the only predictive scores being graded as “fair” using ROC-analysis, [23]. For association with any adverse short-term outcome (PCPC category two to six), the MeningiSSS, the Niklasson Scale, and the Simple Luanda Scale were graded as “poor,” while the Herson–Todd Scale and the Aronin Scale were graded as “failed” [23]. When comparing the predictive scores at their cut-off levels, only the MeningiSSS and the Simple Luanda Scale were close to showing significant differences for death (PCPC category six) or any adverse short-term outcome (PCPC category two to six) (Table 5). Finally, for identifying children later having complications during the hospital stay, none of the predictive scores had an AUC above 0.70; therefore, all predictive scores were graded as either “poor” or “failed” at this task when tested using ROC-analysis [23]. At their respective cut-off level, only the MeningiSSS and the Simple Luanda Scale were significantly associated with occurrence of complications (Table 6).

**Table 5:** Comparison of the predictive scores’ association with short-term outcome

	No.	AUC	Grade	Sens. (%)	Spec. (%)	PPV (%)	NPV (%)	*p*^Cut-off^
Death								
Aronin Scale (%)	101	0.53	Failed	33	83	11	95	0.31
Herson–Todd Scale (%)	88	0.79	Fair	25	92	13	96	0.26
MeningiSSS (%)	77	0.70	Fair	75	71	13	98	0.05
Niklasson Scale (%)	88	0.72	Fair	0	94	0	94	0.57
Simple Luanda Scale (%)^b^	99	0.63	Poor	40	90	18	97	0.04
Any adverse short-term outcome^a^								
Aronin Scale (%)	99	0.56	Failed	27	84	33	80	0.21
Herson–Todd Scale (%)	88	0.53	Failed	18	93	38	83	0.17
MeningiSSS (%)	77	0.63	Poor	44	73	33	81	0.17
Niklasson Scale (%)	88	0.65	Poor	5	94	20	78	0.93
Simple Luanda Scale (%)^c^	98	0.64	Poor	38	81	35	83	0.07

This table shows the Meningitis Swedish Survival Score’s (MeningiSSS) association with short-term outcome compared with existing predictive scores. First, the number of patients included for each predictive score (No.) is shown, followed by the area under the curve (AUC) obtained from the receiver operating curve characteristics analysis together with the performance category grading (Grade) based on this result [23]. Lastly, each predictive score’s sensitivity (Sens.), specificity (Spec.), positive predictive value (PPV), and negative predictive value (NPV) when tested at their cut-off levels using a Chi-square test are shown, including the *p* value of this analysis (*p*^Cut-Off^)

^a^This category comprises of patients with neurological disabilities at discharge defined as Pediatric Cerebral Performance Category Scale (PCPC) category two to five and patient that died (PCPC category six) [22]

^b^In this analysis, the Simple Luanda Scale was tested using the cut-off level stated for predicting death

^c^In this analysis, the Simple Luanda Scale was tested using the cut-off level stated for predicting adverse outcomes

**Table 6:** Comparison of the predictive scores’ ability to identify children later having complications during the hospital stay

	No.	AUC	Grade	Sens. (%)	Spec. (%)	PPV (%)	NPV (%)	*p*^Cut-Off^
All complications^a^								
Aronin Scale (%)	101	0.61	Poor	22	86	61	53	0.28
Herson–Todd Scale (%)	88	0.55	Failed	12	94	63	55	0.34
MeningiSSS (%)	77	0.65	Poor	45	82	71	60	0.01
Niklasson Scale (%)	88	0.66	Poor	9	98	80	51	0.18
Simple Luanda Scale (%)^b^	99	0.68	Poor	33	86	70	58	0.02
Intracerebral injury^c^								
Aronin Scale (%)	101	0.60	Poor	36	85	28	89	0.06
Herson–Todd Scale (%)	88	0.64	Poor	20	92	25	90	0.20
MeningiSSS (%)	77	0.61	Poor	55	73	25	91	0.07
Niklasson Scale (%)	88	0.65	Poor	0	94	0	87	0.38
Simple Luanda Scale (%)^b^	99	0.60	Poor	39	79	22	90	0.16

This table shows the Meningitis Swedish Survival Score (MeningiSSS) ability of identifying children later having complications during the hospital stay, compared with existing predictive scores. First, the number of patients included for each predictive score (No.) is shown, followed by the area under the curve (AUC) obtained from the receiver operating curve characteristics analysis together with the performance category grading (Grade) based on this result [23]. Lastly, each predictive score’s sensitivity (Sens.), specificity (Spec.), positive predictive value (PPV), and negative predictive value (NPV) when tested at their cut-off levels using a Chi-square test are shown, including the *p* value of this analysis (*p*^Cut-Off^)

^a^This category also included intracerebral injury

^b^In this analysis, the Simple Luanda Scale was tested using the cut-off level stated for predicting adverse outcomes

^c^This category included hygroma, generalized brain edema, brain bleeds, ischemic injury, and brain abscess. All intracerebral complications were diagnosed via a brain computer tomography except in three cases: a hygroma diagnosed via repeated brain ultrasounds, an ischemic injury diagnosed via magnetic resonance, and a subarachnoid hemorrhage discovered during postmortem autopsy. Seizures or abnormal neurological findings without any intracerebral structural injury were not included

### MeningiSSS Compared with Individual Risk Factors

Minor correlations were noted when previously known risk factors were tested individually. However, compared with the MeningiSSS, these correlations were weaker.

None of the factors related to patient’s characteristics known predisposing conditions, the child’s age or sex, had any significant association with any primary or secondary outcomes in our study.

Of the three most common causative pathogens, *S. pneumoniae* tended to more often be associated with a worse outcome. Compared with *H. influenzae* and *N. meningitidis*, cases of bacterial meningitis caused by *S. pneumoniae* tended to have higher risk of death (PCPC category six) (10 vs. 5 vs. 0%, *p* = 0.26) and any adverse outcome (PCPC category two to six) (27 vs. 20 vs. 10%, *p* = 0.48).

When testing previously known risk factors related to clinical presentation at admission to the hospital, only three were significantly associated with any adverse outcome (PCPC category two to six); seizures (55 vs. 18%, *p* = 0.01), circulatory shock (60 vs. 20%, *p* = 0.04), and low cerebrospinal fluid (CSF) glucose level (44 vs. 17%, *p* = 0.01). In addition, low CSF glucose level was significantly associated with death (PCPC category six) (22 vs. 2%, *p* < 0.01). None of the remaining factors related to clinical presentation at admission, duration of illness, symptoms, findings in the physical examination, or laboratory findings, showed any significant associations with any primary or secondary outcomes in our study.

## Discussion

Predicting complications and outcome is important; besides, enabling adequate information to patients and relatives, knowing the individual child’s risk can be highly useful when deciding on treatment strategies. In this study, we demonstrated that the new MeningiSSS surpasses existing predictive scores and individual risk factors for several tasks.

### The MeningiSSS Compared with Existing Predictive Scores and Individual Risk Factors

The MeningiSSS performed better than the other tested predictive scores at identifying children later having to be admitted to the intensive care unit or undergo invasive procedures to monitor or manage the intracerebral pressure. In addition, it showed equal or slightly stronger association with occurrence of complications, intracerebral injury, or death compared with the other predictive scores.

The Simple Luanda Scale, designed for use in low- and middle-income countries, is a simple predictive score that uses only five easily assessable criteria [5]. This was the only tested predictive score besides the MeningiSSS which achieved statistical significance at multiple tasks when tested at its cut-off level. However, the Simple Luanda Scale did not perform well in the ROC-analyses. Furthermore, unlike the MeningiSSS, it was unable to identify all cases undergoing an invasive procedure to monitor or manage the intracerebral pressure. The Simple Luanda Scale was originally designed for use in low- and middle-income countries and was validated in similar settings [5]. When resources are limited, its simplicity is a major advantage. In high-income countries where laboratory tests are standard, this predictive score falls short.

The Herson–Todd Scale, the Niklasson Scale, and the Aronin Scale all had major issues excluding them as valid alternatives for clinical use. The Herson–Todd Scale, despite being based on both clinical and laboratory criteria, was graded as “failed” [23] in three out of six ROC-analyses and did not reach statistical significance when tested at its cut-off level in any task. These results are surprising since the Herson–Todd Scale is one of the few predictive scores that has been consistent in previous validation attempts [3–5]. The Herson–Todd Scale was originally developed for bacterial meningitis caused by *Haemophilus influenzae type b* [24]. In our study, this pathogen caused only 40% of cases which is a possible explanation. The Niklasson Scale, besides being graded as “poor” [23] in all ROC-analyses except one, often misclassified cases resulting in higher occurrence of complications in the group classified as low-risk when tested at its cut-off level. The Aronin Scale with only three criteria was the simplest predictive score in our study. However, its abilities were severely lacking, and it had the overall worse results both when tested using ROC-analysis and when tested at its cut-off level.

Of the previously known individual risk factors, causative pathogen, low CSF glucose level, circulatory shock, and seizures at admission to the hospital were all associated with a worse outcome in our study. Others, such as patients’ sex, age, or occurrence of any known predisposing conditions were not. The tendency of individual risk factors to have different levels of impact on outcome in different studies [2] implies that their predictive abilities are less consistent. Although individual risk factors alone provide some information, patients often have multiple risk factors while not fulfilling others, making individual risk factors less useful in clinical practice, when reviewed separately.

### Clinical Use of the MeningiSSS

Predictive scores aiding clinicians in difficult situations are numerous. Examples include Well’s score for deep vein thrombosis and pulmonary embolism together with the CRB-65 for community acquired pneumonia [25–29]. For bacterial meningitis, treatment guidelines facilitate decisions regarding antibiotic treatment and addition of corticosteroids [11–16]. However, less support is provided for decisions regarding invasive procedures, despite involving higher risk for the child [18].

The MeningiSSS, exceling at identifying children later having to be admitted to the intensive care unit or undergo invasive procedures to monitor or manage the intracerebral pressure, could possibly be used to identify high-risk patients already at admission to the hospital. Intracerebral injury caused by rapidly increasing intracerebral pressure is a common cause of death in cases of bacterial meningitis [11–13], and invasive procedures to reduce the intracerebral pressure have been proven effective in reducing the risk of death and neurological disabilities in severe cases of bacterial meningitis [30]. Therefore, identifying these high-risk patients is very desirable and would enable clinicians to prepare and take preventive actions ahead of time. Most importantly, early transfer to a hospital with neurosurgical capability before the child deteriorates hindering transfer could reduce risk of death due to intracerebral injury.

### Strengths and Weaknesses

When creating any new predictive score, overfitting the model is a well-known risk, either by adding several criteria fitting the dataset or testing a vast number of outcomes. In our study, we tried to reduce this risk in two ways: by basing our predictive score on a systematic review of previously identified risk factors and by using a limited number of predefined outcomes. The systematic review investigated general risk factors for overall adverse outcome, neurological sequelae, or death rather than our specific outcome. However, the risk of overfitting should still be reduced despite this disadvantage.

The main strength of this study lies in the standardized data collection process, ensuring that all available data were collected in the same manner. However, as this is a retrospective study, there are also limitations regarding the data. Lack of standardized protocols for clinical examinations and laboratory analyses could result in complications or disabilities being missed, underestimating their occurrence. In addition, a historical material is likely to have a different etiological distribution than today, with a higher occurrence of *H. influenzae* and *S. pneumonia*. However, we did not see any difference in the MeningiSSS ability to predict outcome for different causative pathogens; therefore, this issue should be minor.

Despite being collected retrospectively during a 30-year period, treatment strategies used in our study are comparable to those used in other high-income countries today [11–13]. This allows us to interpret our results in today’s settings and suggests that the MeningiSSS could be suitable for further trials and validation with the aim of future clinical use.

## Conclusions

The MeningiSSS outperformed existing predictive scores at identifying children later having to undergo invasive procedures to monitor or manage the intracerebral pressure in children with bacterial meningitis. Our results need further external validation before use in clinical practice. Thus, the MeningiSSS could potentially be helpful when making difficult decisions concerning intracerebral pressure management.
